# Validation of the Brazilian Version of the Cambridge Renal Stone Patient-Reported Outcome Measure (Br-CReSP) versus a Generic Questionnaire for Assessing Health-Related Quality of Life in Nephrolithiasis

**DOI:** 10.1590/S1677-5538.IBJU.2025.0553

**Published:** 2025-11-20

**Authors:** Alexandre Danilovic, Daniel Gabriele Sucupira, Oliver Wiseman, Elaine Brasil, Fabio Cesar Miranda Torricelli, Giovanni Scala Marchini, Carlos Batagello, Rodrigo Perrela, Fabio Carvalho Vicentini, William C. Nahas, Eduardo Mazzucchi

**Affiliations:** 1 Universidade de São Paulo Faculdade de Medicina Hospital das Clínicas HCFMUSP São Paulo SP Brasil Departamento de Urologia, Hospital das Clínicas HCFMUSP, Faculdade de Medicina, Universidade de São Paulo, São Paulo, SP, Brasil; 2 Universidade de São Paulo Faculdade de Medicina Hospital das Clínicas HCFMUSP São Paulo SP Brasil Departamento de Urologia, Hospital das Clínicas HCFMUSP, Faculdade de Medicina, Universidade de São Paulo, São Paulo, SP, Brasil; 3 Cambridge University Addenbrooke's Hospital Department of Urology Cambridge United Kingdom Department of Urology, Addenbrooke's Hospital, Cambridge University, Cambridge, United Kingdom

**Keywords:** Nephrolithiasis, Kidney Calculi, Quality of Life

## Abstract

**Purpose:**

No validated tool specifically assesses health-related quality of life (HRQoL) in Brazilian patients with kidney stones. The Cambridge Renal Stone Patient-Reported Outcome Measure (CReSP) is a self-administered questionnaire that evaluates the impact of kidney stones on patients’ QoL over the preceding seven days. This study aimed to translate the CReSP into Portuguese, validate it, and compare it with the validated generic SF-12 questionnaire.

**Materials and Methods:**

The CReSP questionnaire was translated into Portuguese following Guillemin's guidelines. Patients with and without kidney stones completed the Brazilian version of the CReSP (Br-CReSP) and SF-12 questionnaires. Internal consistency, test-retest reliability, discriminant validity, and convergent validity with SF-12 components were evaluated. Logistic regression assessed the discriminant capacity of Br-CReSP and SF-12 components for nephrolithiasis.

**Results:**

One hundred patients completed both questionnaires. Internal consistency was high across all domains and the total score (Cronbach's α = 0.92). Test-retest reliability demonstrated strong correlations for all domains and the total score (ICC = 0.94). Discriminant validity was evidenced by significant differences between patients with and without kidney stones, with large effect sizes. Convergent validity was shown by significant inverse correlations between the Br-CReSP and SF-12 (p < 0.001). The Br-CReSP outperformed PCS-12 and MCS-12 in predicting nephrolithiasis (AUC = 0.91 vs. 0.84 and 0.73, respectively).

**Conclusions:**

The validated Br-CReSP outperforms SF-12 in assessing HRQoL in Brazilian patients with kidney stones.

## INTRODUCTION

Nephrolithiasis, a prevalent urological condition, significantly impairs health-related quality of life (HRQoL) due to acute pain, transient disability, and, in severe cases, renal function loss (1-6). Its incidence varies globally, influenced by geographic, climatic, ethnic, dietary, and genetic factors (7). With recurrence rates reaching up to 50% within five years, nephrolithiasis imposes a substantial and recurrent burden on patients’ daily functioning (8-10).

Current outcome measures for nephrolithiasis primarily emphasize stone-free rates (SFR) and complications, often overlooking patient-centered outcomes such as HRQoL (11). Evidence on HRQoL in nephrolithiasis treatment remains limited, and neither the European Association of Urology (EAU) nor the American Urological Association (AUA) guidelines currently integrate HRQoL assessments into treatment decision-making (12, 13). Incorporating HRQoL data through validated questionnaires can standardize and quantify patients’ physical and psychological well-being, fostering shared decision-making and aligning with patient-centered care principles (14, 15).

Patient-reported outcome measures (PROMs) are validated instruments designed to capture patient's perspective on disease impact (16). The Cambridge Renal Stone Patient-Reported Outcome Measure (CReSP) is a disease-specific PROM comprising 14 questions across six domains — pain, urinary symptoms, work and daily activities, anxiety, and dietary changes, and overall quality of life — scored on a Likert scale, with higher scores indicating worse HRQoL (17). Unlike other HRQoL tools, the CReSP is tailored to kidney stone patients and focuses on symptom burden over the preceding seven days, making it uniquely suited for evaluating treatment outcomes in nephrolithiasis.

We hypothesized that a disease-specific questionnaire for assessing HRQoL in patients with kidney stones would provide greater accuracy than a generic questionnaire, enabling urologists to better understand patient needs and enhance clinical practice. This study aimed to translate and validate the CReSP into Brazilian Portuguese (Br-CReSP), ensuring linguistic and conceptual equivalence while preserving its psychometric robustness. Additionally, we compared the disease-specific Br-CReSP with the generic SF-12 questionnaire to assess their relative performance in evaluating HRQoL in Brazilian patients with nephrolithiasis.

## MATERIALS AND METHODS

### Study design and participants

This prospective study was conducted at a specialized public university hospital, enrolling native Portuguese-speaking patients aged 18 years or older, with or without kidney stones. All participants provided written informed consent. Exclusion criteria included ureteral stones, other urological conditions, pelvic pain syndrome, use of anticholinergics, alpha-blockers, calcium channel blockers, or phosphodiesterase type 5 inhibitors, illiteracy, psychiatric disorders, or age under 18 years. Data collection occurred between December 2022 and January 2024, adhering to the Declaration of Helsinki. The study was approved by the institutional review board (IRB approval number: 83672324.7.0000.0068).

### Translation and adaptation of the CReSP questionnaire

The Cambridge Renal Stone Patient-Reported Outcome Measure (CReSP) was translated into Brazilian Portuguese (Br-CReSP) following established guidelines for cross-cultural adaptation. Two independent, native Portuguese-speaking urologists performed the initial translation. A consensus meeting with the study authors resolved discrepancies. An independent bilingual professional back-translated the questionnaire into English, and the original CReSP author reviewed both versions to ensure conceptual equivalence, with further consensus meetings addressing any discrepancies (Supplementary material 1).

### Data Collection

Participants completed the self-administered Br-CReSP and the validated Brazilian Portuguese SF-12 questionnaire (version 1.0, public domain) (18). The SF-12, a shortened version of the short Form 36, comprises two components: the Physical Component Score (PCS-12) and the Mental Component Score (MCS-12), with higher scores indicating better quality of life, in contrast to the Br-CReSP, where higher scores reflect worse HRQoL. To assess temporal stability, participants completed the Br-CReSP twice, with a seven-day interval.

### Statistical Analysis

Statistical analyses were performed using JASP software (version 0.18.3). Confirmatory Factor Analysis was conducted to evaluate the Br-CReSP's internal structure based on the model by Ragab et al. (17). Internal consistency was assessed using Cronbach's alpha for the total score and individual domains of Br-CReSP, with α ≥ 0.70 considered acceptable. Temporal stability was evaluated using Spearman's correlation coefficient for test-retest reliability, with coefficient interpreted as low (± 0.1), moderate (± 0.3), or strong (± 0.5). The Blant-Altman method assessed agreement between test and retest measurements.

Discriminant validity was evaluated by comparing Br-CReSP mean scores between patients with kidney stones and controls using independent sample t-tests. Levene's test assessed variance homogeneity, and Welch's statistic was applied when necessary. Bootstrapping (1,000 resamplings; 95% Bias-Corrected and accelerated confidence intervals) was used to address non-normal distributions and enhance result reliability (19). Effect sizes were categorized as small (0.20 - 0.49), medium (0.50 - 0.79), or large (≥0.80).

Convergent validity was assessed by calculating Spearman's correlation coefficient between Br-CReSP total score and the PCS-12 and MCS-12 scores of the SF-12. To compare the predictive performance of the Br-CReSP, PCS-12, and MCS-12 for kidney stones, logistic regression models were fitted for each tool, adjusted using the Wald test. Performance metrics, including area under the curve (AUC), accuracy, sensitivity, specificity, and precision, were calculated. A p-value < 0.05 was considered statistically significant.

## RESULTS

### Participant Characteristics

Demographic and clinical features of study population are presented in Table-1. A total of 100 patients completed both the Br-CReSP and SF-12 self-administered questionnaires. Of these, 56% were female, 66% were Caucasians, and 67% were employed. Kidney stones were present in 70 (70%) participants, with 41 (58.6%) of these reporting a previous stone event.

**Table 1:** Demographic and clinical features of the study population.

Feature		Respondents without kidney stones (N=30)	Respondents with kidney stones (N=70)
Age, years, mean (SD)		50.30 (13.21)	54.21(10.14)
Female gender, N (%)		14 (46.67)	42 (60.00)
**Marital status, N (%)**			
	Single	7 (23.33)	18 (25.71)
	Married	19 (63.33)	40 (57.14)
	Window	2 (6.67)	4 (5.71)
	Divorced	1 (3.33)	6 (8.57)
	Missing	1 (3.33)	1 (1.43)
	Other	0 (0.00)	1 (1.43
**Ethnicity, N (%)**			
	Caucasian	20 (66.67)	46 (65.71)
	African American	3 (10.00)	13 (18.57)
	More than one race	7 (23.33)	11 (15.71)
**Educational level, N (%)**			
	Incomplete	0 (0.00)	11 (15.71)
	Elementary school	8 (26.67)	17 (24.29)
	High school	6 (20.00)	32 (45.71)
	University graduate	6 (20.00)	7 (10.00)
	Postgraduation	10 (33.33)	3 (4.29)
**Ocupation, N (%)**			
	Working	26 (86.67)	41 (58.57)
	Unemployed	0 (0.00)	6 (8.57)
	Retired	1 (3.33)	15 (21.43)
	Housewife	3 (10.00)	8 (11.43)
**Stone event, N (%)**			
	No	30 (100.00)	29 (41.43)
**Previous treatment, N (%)**			
	Medical expulsive therapy	1 (3.33)	24 (34.29)
	Ureteroscopy	3 (10.00)	8 (11.43)
	Shockwave lithotripsy	1 (3.33)	20 (28.57)
	Percutaneous nephrolithotomy	2 (6.67)	13 (18.57)
	No treatment	23 (76.67)	5 (7.14)

SD = Standard deviation

### Validation

Descriptive statistics for the 14 items of the Br-CReSP are provided in Table-2. No univariate inconsistencies were detected. Confirmatory factor analysis confirmed an adequate fit for the six-factor structure of the Br-CReSP. Items related to pain and anxiety about pain yielded the highest scores, indicating their significant impact on patient's health-related quality of life.

**Table 2:** Descriptive statistics for the 14 items of Br-CReSP.

Descriptor	Mean	Standard Deviation	Variance	Skewness	Kurtosis
1. How much did pain interfere with your day-to-day activities?	2.12	1.37	1.86	0.85	-0.66
2. How much did pain interfere with your enjoyment of life?	2.06	1.43	2.06	0.98	-0.57
3. how much did you worry about pain?	4.70	3.77	14.21	0.35	-1.58
4. I have had blood in my urine	1.55	1.12	1.26	2.06	3.11
5. I have nausea	1.61	1.02	1.05	1.71	2.30
6. I have trouble doing all my usual work include work at home	2.07	1.35	1.82	0.82	-0.77
7. I have trouble doing all my regular leisure activities with others	1.95	1.31	1.72	1.13	-0.05
8. I have trouble doing all my family activities that I want to do	1.92	1.28	1.65	1.09	-0.20
9. I felt fearful	2.22	1.45	2.09	0.75	-0.84
10. I found it hard to focus on anything over than my anxiety	2.01	1.28	1.63	0.97	-0.28
11. My worries overwhelmed me	2.47	1.50	2.25	0.47	-1.23
12. I am bothered by side effects of treatment	1.78	1.24	1.53	1.45	0.98
13. How much have you been bothered by recommended alterations to your fluid intake?	1.63	1.06	1.12	1.73	1.93
14. How much have dietary of fluid changes affected your daily life?	1.62	1.07	1.15	1.72	1.83

### Internal Consistency

Internal consistency was robust across the Br-CReSP domains and total score: pain (α = 0.91, 95% CI [0.86-0.96]), work and daily activities (α = 0.94, 95% CI [0.91-0.97]), anxiety (α = 0.85, 95% CI [0.80-0.91]), dietary changes (α = 0.82, 95% CI [0.72-0.93]), and total score (α =0.92, 95% CI[0.90-0.94]). Average inter-item correlations were 0.84 for pain, 0.84 for work and daily activities, 0.60 for anxiety, 0.70 for dietary changes, and 0.55 for the total score, all deemed satisfactory. Cronbach's alpha could not be calculated for single-item domains (urinary symptoms and intestinal symptoms); however, these domains contribute to the overall validity of the instrument.

### Convergent validity

Convergent validity was assessed using Spearman's correlation between the Br-CReSP total score and the SF-12 components. As expected, significant negative correlations were observed with the PCS-12 (r = −0.61, p < 0.001) and MCS-12 (r = −0.44, p < 0.001), confirming the Br-CReSP's alignment with established HRQoL measures.

### Discriminant validity

Welch's statistic revealed significant differences in all Br-CReSP domains between patients with and without kidney stones (Supplementary material 2). Scores were consistently higher in the kidney stone group, with large effect sizes, demonstrating the Br-CReSP's ability to discriminate between groups based onHRQoL. Logistic regression models predicting nephrolithiasis demonstrated superior performance for the Br-CReSP compared to PCS-12 and MCS-12, with higher accuracy (0.86 vs. 0.74 vs. 0.68), ACU (0.91 vs. 0.84 vs. 0.73), sensitivity (0.86 vs. 0.79 vs. 0.86), specificity (0.87 vs. 0.63 vs. 0.27), and precision (0.94 vs. 0.83 vs. 0.73), respectively.

### Temporal Stability

**Figure 1:**
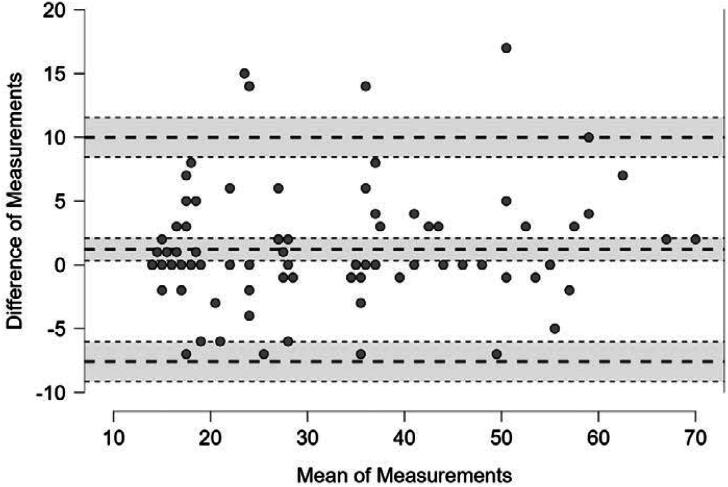
Bland-Altman scatter plot. The X-axis shows the mean of test and retest scores for each patient, while the Y-axis shows the difference between the two measurements. A horizontal dotted line close to 0 represents the mean difference (bias) between the test and retest measurements [1.21 (95% CI 0.32 - 2.10). The two horizontal dotted lines above and below the mean difference dotted line represent the 95% limits of agreement: −7.58 (95% CI −9.13 - −6.04) and 10.00 (95% CI 8.46 - 11.55).

Test-retest reliability, assessed over seven-day interval, showed strong Spearman's correlations for all Br-CReSP domains and total score indicating temporal stability. The Bland-Altman analysis (Figure-1) revealed a low mean difference between test and retest scores (1.21, 95% CI [0.32 - 2.10]), with most data points within the limits of agreement, confirming the Br-CReSP's stability across varying patient scores.

## DISCUSSION

This study presents the first translation and validation of the Cambridge Renal Stone Patient-Reported Outcome Measure into Brazilian Portuguese, establishing a disease-specific tool for assessing HRQoL in patients with kidney stones. The Br-CReSP demonstrated superior accuracy, AUC, sensitivity, specificity, and precision compared to the generic SF-12 questionnaire, highlighting its enhanced suitability for evaluating HRQoL of patients with kidney stones. As a comprehensive, disease-specific instrument, the Br-CReSP effectively captures the patient's perspective on the impact of nephrolithiasis, offering a valuable tool for clinical and research applications.

Incorporating HRQoL assessment into the evaluation of nephrolithiasis outcomes is essential, as it provides insights beyond traditional metrics such as SFR and complications (1, 6, 20). While generic instruments like the Short Form 36 are widely used across medical conditions, they lack the specificity required to accurately monitor HRQoL in kidney stone patients (21). The Br-CReSP addresses this gap by offering a tailored approach to capture the unique burdens of nephrolithiasis.

The psychometric robustness of the Br-CReSP was confirmed through rigorous validation. High internal consistency across all domains (Cronbach's α ≥ 0.82) and strong test-retest correlations demonstrated its reliability and temporal stability. Convergent validity was established though significant inverse correlations with the SF-12 components. Discriminant validity was evidenced by significant differences in Br-CReSP domain and total score between patients with and without kidney stones, with large effect sizes underscoring its construct validity. Notably, the Br-CReSP outperformed the SF-12 in discriminating nephrolithiasis, supporting its adoption in clinical practice for precise HRQoL assessment.

The Wisconsin Stone Quality of Life (WISQOL) questionnaire is another disease-specific PROM for nephrolithiasis (22). However, a retrospective multicenter study found no association between SFR post-surgical intervention and improved HRQoL using WISQOL (23). Both WISQOL and CReSP demonstrated improvement in scores for patients opting for surgery over observation (24). While WISQOL assesses the broader burden of urinary stone disease, the CReSP focuses specifically on kidney stones and their impact over the preceding seven days, making it particularly suited for evaluating acute treatment effects, such as post-ureteroscopy pain, which significantly affects HRQoL in the first seven postoperative days (25).

A key strength of this study is its rigorous validation methodology, in a diverse population, including direct comparisons of discriminant capacity with a generic questionnaire, an original contribution to the literature. This approach extends beyond the original CReSP validation study (17), reinforcing the Br-CReSP's utility as a disease-specific HRQoL tool.

However, limitations include the single-center design and lack of longitudinal assessment. Future research should evaluate the Br-CReSP responsiveness to treatment interventions and compare it with other PROMs like WISQOL. Additionally, exploring correlations between Br-CReSP scores and objective clinical outcomes, such as SFR and complication rates, would further validate its role in guiding treatment decisions.

## CONCLUSIONS

The Br-CReSP is the first validated, disease-specific PROM for assessing HRQoL in Brazilian patients with kidney stones. It addresses a critical gap in patient-centered outcome evaluation by providing a reliable and precise tool tailored to nephrolithiasis. The Br-CReSP's superior psychometric properties and discriminant capacity compared to generic instruments like the SF-12 underscore its potential to enhance clinical practice. Future studies should explore its utility in guiding treatment decisions and investigate correlations between Br-CReSP sores, SFR, and complication rates to further integrate HRQoL into evidence-based management of nephrolithiasis.

## Data Availability

Uninformed
