# Supplementary material for: Validation of the Brazilian Version of the Cambridge Renal Stone Patient-Reported Outcome Measure (Br-CReSP) versus a Generic Questionnaire for Assessing Health-Related Quality of Life in Nephrolithiasis
Source: Int Braz J Urol. 2025 Nov 20;52(2):e20250553. doi: 10.1590/S1677-5538.IBJU.2025.0553 (PMC13124181; doi:10.1590/S1677-5538.IBJU.2025.0553)
Supplement: Supplementary file 1 [file 1677-6119-ibju-52-02-e20250553-Suppl1.pdf]

APPENDIX

Supplementary material 1 - CReSP questionnaire.

Thank you for agreeing to complete this form.
This will help us to understand the impact that your kidney stone has on your life.

Please respond to each question or statement by marking one box per row.

**Pain**	**1**	**2**	**3**	**4**	**5**
During the past 7 days	Not at all	A little bit	Somewhat	Quite a bit	Very much
1. How much did pain interfere with your day to day activities?					
2.How much did pain interfere with your enjoyment of life?					

**Pain**	**1**	**2**	**3**	**4**	**5**	**6**	**7**	**8**	**9**	**10**
During the past 7 days	Not at all									Very much
3.How much did you worry about pain?										

**Urinary Symptoms:**	**1**	**2**	**3**	**4**	**5**
During the past 7 days	Not at all	A little bit	Somewhat	Quite a bit	Very much
4.I have had blood in my urine					

**GIT Symptoms:**	1	2	3	4	5
During the past 7 days	Not at all	A little bit	Somewhat	Quite a bit	Very much
5.I have nausea					

**Work, daily activities and travel plans**	**1**	**2**	**3**	**4**	**5**
During the past 7 days	Never	Rarely	Sometimes	Often	Always
6.I have trouble doing all of my usual work (include work at home)?					
7. I have trouble doing all of my regular leisure activities with others					
8.I have trouble doing all of the family activities that I want to do					

**Anxiety**	**1**	**2**	**3**	**4**	**5**
During the past 7 days	Never	Rarely	Sometimes	Often	Always
9.I felt fearful					
10. I found it hard to focus on anything other than my anxiety					
11. My worries overwhelmed me					
12. I am bothered by side effects of treatment					

**Dietary changes:**	**1**	**2**	**3**	**4**	**5**
During the past 7 days	Not at all	A little bit	Somewhat	Quite a bit	Very much
1. How much have you been bothered by recommended alterations to your fluid intake?					
2.How much have dietary or fluid changes affected your daily life?					

Thank you for completing this for

Supplementary material 2 - Evidence of discriminant validity.

	Nephrolithiasis		Levene						95% CI for Cohen's d	
**Domain**	**No (n=30)**	**Yes (n=70)**	**F**	**t**	**df**	**Lower**	**Upper**	**Cohen's d**	**Lower**	**Upper**
Pain	2.20 0.76	5.03 ± 2.77	68.54*	-7.87	88.87*	-3,514	-2,082	-1.39	-1.86	-0.91
Urinary Symptoms	1.03 ± 0.18	1.77 ± 1.28	45.00*	-4.73	75.34*	-1,046	-0,445	-0.81	-1.25	-0.36
Work and Daily Activities	3.30 ± 1.29	7.07 ±3.87	48.81*	-7.27	94.35*	-4,706	-2,728	-1.31	-1.77	-0.84
Anxiety	4.97 ± 1.71	9.99 ± 4.59	26.50*	-7.95	96.80*	-6,305	-3,744	-1.45	-1.92	-0.97
Dietary changes	2.23 ± 1.10	3.69 ± 2.10	25.05*	-4.52	93.75*	-2,064	-0,766	-0.87	-1.31	-0.42
Total Score	16.43 ± 4.59	35.40 ± 15.40	38.35*	-9.38	91.26*	-22,957	-14,855	-1.67	-2.16	-1.17

*p < 0.001

F = the test statistic for Levene’s test. Larger values indicate greater evidence against the null hypothesis of variances; t = t-statistic; df = degrees of freedom
